# Fiberoptic Bronchoscopic Findings in Patients Suffering from Emerging Pulmonary Lophomoniasis: A First Registry-Based Clinical Study

**DOI:** 10.1155/2022/8034295

**Published:** 2022-06-16

**Authors:** Masoud Aliyali, Amirmasoud Taheri, Mahdi Fakhar, Ali Sharifpour, Maryam Nakhaei, Siavash Abedi, Hossein Mehravaran, Sepideh Safanavaei

**Affiliations:** ^1^Toxoplasmosis Research Center, Communicable Diseases Institute, Iranian National Registry Center for Lophomoniasis (INRCL), Mazandaran University of Medical Sciences, Sari, Iran; ^2^Pulmonary and Critical Care Division, Imam Khomeini Hospital, Mazandaran University of Medical Sciences, Sari, Iran

## Abstract

**Background:**

*Lophomonas blattarum* is an emerging protozoan agent that mainly infects the lower respiratory system, causing pulmonary lophomoniasis. The bronchoscopic findings in patients with pulmonary lophomoniasis have not been investigated yet. Accordingly, we assess the bronchoscopic findings of lophomoniasis in patients suffering from pulmonary lophomoniasis through a registry-based clinical study.

**Methods:**

In this retrospective study, of 480 patient candidates for bronchoscopy, 50 *Lophomonas*-positive patients were enrolled. Demographic data, relevant characteristics, and bronchoscopy findings of the patients were recorded and analyzed.

**Results:**

Overall, 50 (male = 32, female = 18) patients with an average age of 61.8 ± 13.3 years were examined. Nineteen patients (38%) had normal bronchoscopic findings, and 31 patients (62%) had abnormal bronchoscopic findings. According to the severity index, most (52%) of patients had mild severity, followed by moderate (30%) and severe (18%) cases. The highest involvement was in the right lung bronchus (46%), and the lowest was in the carina (8%). Furthermore, purulent and mucosal secretions in the right and left lung bronchus were the most abnormalities found in different anatomical locations.

**Conclusion:**

For the first time, the current study demonstrated that pulmonary lophomoniasis does not have pathognomonic bronchoscopic findings. However, each suspected patient must be checked for lophomoniasis, even with normal bronchoscopic findings, particularly in endemic areas.

## 1. Introduction

Lophomoniasis is an emerging parasitic disease caused by *Lophomonas blattarum* (*L. blattarum*). The protozoan parasite infects the upper and lower respiratory systems, causing pulmonary lophomoniasis [1, 2]. It is found in the intestines of insects like cockroaches [2–4]. It was initially discovered in 1911 via light microscopy [5]. This parasite facilitates food digestion in the intestine of its reservoir host. By excreting *Lophomonas* in their feces, these insects contaminate the human environment [2–4]. Humans are infected by inhaling the aerosol, which contains cysts. Numerous tissues and organs, including the sinuses, lungs, respiratory tract, and genitourinary tract, become dysfunctional because of the establishment of this parasite. The most frequent symptoms among the patients included low-grade fever, chronic cough, and sputum. Radiographic imaging of the lungs may show signs of pneumonia, bronchitis, pulmonary abscess, and pleural effusion [1, 6]. Light microscopy and, more recently, polymerase chain reaction (PCR) techniques are used to diagnose this parasite using a sample taken from bronchoalveolar lavage fluid (BALF) [4, 7].

Fiberoptic bronchoscopy (FOB) is a crucial technique for identifying and controlling a variety of lung diseases [8], including lophomoniasis [9]. There are several case reports of lophomoniasis, mainly from Asia, including China and Iran [1, 2, 10], but none of them studied the bronchoscopic view of these cases. Thus, the bronchoscopic findings in patients with pulmonary lophomoniasis have not been investigated yet. Accordingly, we investigate the bronchoscopic findings in patients referred to the Iranian National Registry Center for Lophomoniasis (INRCL) to explore a possible pathognomonic view of this disease.

## 2. Subjects and Methods

### 2.1. Study Population

In this retrospective study, 50 *Lophomonas*-positive patients were enrolled in our study population of 480 patient candidates for FOB at Imam Khomeini Hospital between 2017 and 2021. The inclusion criteria were patients with age over 18 years.

### 2.2. Fiberoptic Bronchoscopy (FOB)

A flexible FOB examination was done for all patients (*n* = 480) who were bronchoscopy candidates in a fully sterile condition in the bronchoscopy room or operation room. Wedging the bronchoscope's tip into the nondependent lobes, particularly the middle lobe of the right lung and the lingula of the left lung, provided a BALF specimen. The lesion images with the largest radiologic abnormalities were utilized to define the lobe used for BALF collection. 5–20 mL of sterile normal saline was instilled 2–4 times, divided into 5–20 vials. To extract the saline, gentle manual suction was used. BALF specimens were collected in sterilized containers and sent to the INRCL laboratory at Imam Khomeini hospital in 2–5 mL samples within 1–3 hours at room temperature.

### 2.3. Microscopic Examination

**Figure 1:**
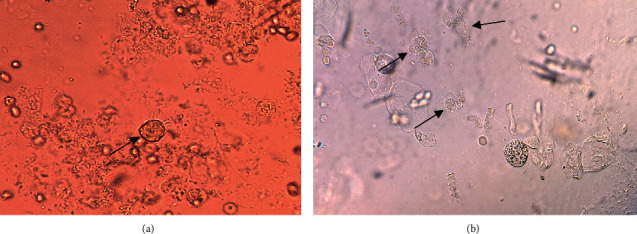
Direct smear of the bronchoalveolar lavage fluid (BALF) specimen with mild (a) and moderate (b) severity indices represents *Lophomonas* trophozoite (arrow ahead).

The BALF specimens were spun at 500 g for 2 minutes. After that, the sedimentation was smeared onto a glass slide and studied under a light microscope for the existence of *Lophomonas* spp. We also used a light microscope to calculate the density of *Lophomonas*. A novel severity index (SI) for *Lophomonas* infection was graded as mild to severe parasite density by measuring parasites per high power microscopic fields (HPFs) derived from the experience in INRCL (X400). As a result, mild density was defined as 1–10 parasites/100HPF, moderate density as 1–10 parasites/10HPF, and severe density as 1–10 parasites/HPF [9] (Figure 1).

### 2.4. Data Analysis

We use percent and frequency for qualitative (gender, smoking status, underlying disease, comorbidity, and bronchoscopic anatomical location) variables, and for quantitative (age) variables, we use mean and standard deviation. A *p* value of less than 0.05 was regarded as statistically significant. Demographic data were analyzed by IBM SPSS version 26. All bronchoscopic reports were collected and categorized into four anatomical locations: trachea, carina, right bronchus, and left bronchus.

## 3. Results

### 3.1. Demographics and Lab Findings

According to the inclusion criteria, 50 individuals (male = 32, female = 18) with a mean age of 61.8 ± 13.3 years were included in this study. The male gender has a statistically significantly higher incidence (*p*=0.001). Patients in this study were divided into six different age groups. The highest frequency of patients was in the age range of 61–70 years, with 19 patients (38%), and the lowest was in the age group of 20–30 years (*p*=0.02). The other variables and their correlation are shown in Table 1.

According to SI of *Lophomonas* infection, most of patients (52%) had mild severity, followed by moderate (30%), and severe (18%) cases. Smoking status was the only factor which was statistically related to mild and moderate severity (*p* < 0.05). The other variables and their correlation are shown in Table 2.

### 3.2. Bronchoscopic Findings

**Figure 2:**
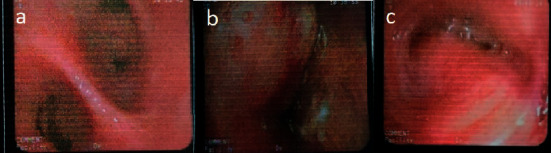
Bronchoscopic view of *Lophomonas*-positive patients. (a) Purulent secretion view in the carina. (b) Masslike lesion view in the right lung bronchus. (c) Mucosal secretions view in the left lung bronchus.

Bronchoscopic findings of patients with *Lophomonas* infection were classified into four sections: trachea, carina, right lung bronchus, and left lung bronchus based on anatomical location. Of 50 patients, 19 (38%) had normal views and 31 (62%) had abnormal views. The observed views by anatomical locations and frequency are shown in Table 2. The highest involvement was in the right lung bronchus (46%), and the lowest involvement was in the carina (8%). Among the views seen in all anatomical locations, most were related to purulent and mucosal secretions (see Table 3) (Figure 2).

## 4. Discussion

In our study, 50 patients were enrolled based on the inclusion criteria, of 480 candidates for bronchoscopy, whose *L. blattarum* infection was confirmed based on the lavage sample taken from them. The majority of the patients in our study were men, which is consistent with the findings of earlier investigations [1–3, 9]. This result can be considered from a variety of angles. As we all know, men and women are behaviorally, socially, and biologically distinct [11]. These disparities can be linked to men's higher exposure to the outside environment than women's [12, 13] as well as testosterone's effect on most parasite diseases [14–16].

The age range of 61–70 years was associated with the highest rate of involvement in our study, which was in line with the findings of other studies [1, 2, 9]. However, multiple studies have reported the presence of *L. blattarum* infection in children [17–20], indicating the possibility of two age peaks of disease, which could be explained by the altered immune systems of these two age groups [21].

Given that the vast majority of the patients in our study were nonsmokers and had a mild or moderate form of infection, and that this correlation was also statistically significant, it is inferred that not smoking increases the risk of the disease. Because there is extensive and countless research on the effect of smoking on raising the prevalence of respiratory illnesses, this result should be thoroughly examined [22, 23]. Our results can be attributed to the small number of samples in our analysis, as well as the hypothesis that smoking prevents protozoan implantation by damaging the airway epithelium [24–26], making smokers less likely to get the infection.

Patients in this study had a variety of underlying medical conditions. Still, there was no statistical correlation between them and *L. blattarum* infection, which has never been studied in other research and could be due to our patients' smaller sample size. Considering that our patients' underlying conditions, such as diabetes, cancer, Cushing's disease, and others, impair the immune system [27–30], and *L. blattarum* has been reported in patients with compromised immune systems [6], further research is needed to study better the link between underlying illnesses and *Lophomonas* infection.

The only comorbidities observed in the patients we studied were tuberculosis and coronavirus disease 2019 (COVID-19). Considering that both of these diseases cause pulmonary involvement, and a case of tuberculosis [31] and COVID-19 [32] coinfection with lophomoniasis has been reported, it is acceptable to further evaluate the prevalence of lophomoniasis and these comorbidities. No research has been conducted on the bronchoscopic findings of *Lophomonas* infection in adults with this sample size to see whether a possible specific view is pathognomonic for the disease.

Of the 50 patients included in the study, 38% of patients had normal views on bronchoscopy and most patients had abnormal views in all four anatomical regions, of which the most common manifestation was purulent-mucosal secretion. As we know, purulent-mucosal discharge is a natural and nonspecific response seen in most respiratory infections [33].

The right lung bronchus was found to have the highest frequency of abnormal views in this study. The protozoan is more likely to invade the right lung since the disease is spread through airborne particles and the main bronchus of the right lung is less angular to the carina. However, this concept has been suggested to be investigated further in a separate study with two lavage samples (both from the left lung and the right lung). Another unexpected finding was cavities in a patient's left lung who had no underlying disease and no history of drug abuse [34]. This patient also had a severe form of *L. blattarum* infection, which suggests that a severe form of the infection could appear in ways beyond our imagination.

The disease was categorized into three groups: mild, moderate, and severe. None of the variables, including gender, age group, underlying condition, and concomitant disease, were associated with disease severity except smoking status, which was statistically associated with mild and moderate forms of the disorder. As previously stated, nonsmokers were significantly associated with mild and moderate types of lophomoniasis.

In conclusion, our study showed that *L. blattarum* infection did not have pathognomonic bronchoscopic findings. However, even in patients with normal bronchoscopic findings, BALF samples should be obtained in lophomoniasis suspected patients . We actually believe that we know very little about this new protozoan, and many aspects of it are unknown. Furthermore, *Lophomonas* has only been reported from China, India, Mexico, Peru, and Iran, with nearly no European or North American countries reporting this protozoan to the best of our knowledge. Because the prevalence of the cockroaches that transmitted the protozoan is almost high in all countries, and in order to prevent irreversible complications of this protozoan such as lung cavities, it is necessary to conduct such studies to identify different aspects of these emerging protozoa. As a whole, we advised the pulmonologists, particularly in endemic regions, that each bronchoscopy candidate must be screened to rule out lophomoniasis.

### 4.1. Limitation

The low number of participants and lack of a control group were the most important limitations of our study.

## Figures and Tables

**Table 1:** Demographic findings and characteristics of patients with *Lophomonas* infection referred to INRCL.

Characteristic	*Lophomonas*-positive patients (%)	*p* value
Age		
20–30	1 (2)	0.02^*∗*^
31–40	2 (4)	
41–50	7 (14)	
51–60	7 (14)	
61–70	19 (38)	
>70	14 (28)	

Gender		
Male	32 (64)	0.001^*∗*^
Female	18 (36)	

Smoking status		
Smoker	14 (28)	0.001^*∗*^
Nonsmoker	36 (72)	

Underlying disease		
Diabetes mellitus	5 (10)	0.31
Cancer	3 (6)	
Asthma	2 (4)	
Cushing's	1 (2)	
Epilepsy	1 (2)	
Hypertension	3 (6)	
Rheumatoid arthritis	1 (2)	

Comorbidity		
Tuberculosis	2 (4)	0.21
COVID-19	1 (2)	

Severity index		
Mild	26 (52)	0.03^*∗*^
Moderate	15 (30)	
Severe	9 (18)	

Total	50	

^*∗*^indicates significant value (*P* < 0.05).

**Table 2:** Demographic findings and characteristics of patients with *Lophomonas* infection referred to the INRCL according to the severity index.

Characteristic	Mild (%)	Moderate (%)	Severe (%)	Total
Gender				
Male	16 (50)	10 (31.25)	6 (12)	32
Female	10 (55.5)	5 (27.7)	3 (16.8)	18
*p* value	>0.05	>0.05	>0.05	

Age				
20–30	0 (0)	0 (0)	1 (2)	1
31–40	1 (2)	0 (0)	1 (2)	2
41–50	3 (6)	2 (4)	2 (4)	7
51–60	3 (6)	2 (4)	2 (4)	7
61–70	9 (18)	8 (16)	2 (4)	19
>70	10 (20)	3 (6)	1 (2)	14
*p*value	>0.05	>0.05	>0.05	

Underlying disease				
Diabetes mellitus	1 (2)	1 (2)	3 (6)	5
Cancer	2 (4)	1 (2)	0 (0)	3
Asthma	0 (0)	1 (2)	1 (2)	2
Cushing's	0 (0)	1 (2)	0 (0)	1
Epilepsy	1 (2)	0 (0)	0 (0)	1
Hypertension	1 (2)	1 (2)	1 (2)	3
Rheumatoid arthritis	0 (0)	1 (2)	0 (0)	1
*p* value	>0.05	>0.05	>0.05	

Comorbidity				
Tuberculosis	2 (4)	0 (0)	0 (0)	2
COVID-19	0 (0)	0 (0)	1 (2)	1
*p* value	>0.05	>0.05	>0.05	

Smoking status				
Yes	7 (14)	4 (8)	3 (6)	14
No	19 (38)	11 (22)	6 (12)	36
*p* value	<0.05^*∗*^	<0.05^*∗*^	>0.05	50

^*∗*^Significant value (*P* < 0.05).

**Table 3:** Frequency of bronchoscopic findings of patients with *Lophomonas* infection referred to the INRCL according to the anatomical locations.

Anatomical locations	Bronchoscopic findings	Frequency (%)
Trachea	Normal	43 (84.32)
	Pleural secretion	3 (5.88)
	Malacia	2 (3.92)
	Erythema	1 (1.96)
	Stenosis	2 (3.92)

Carina	Normal	(92) 46
	Widening	(4) 2
	Pleural secretion	(4) 2

Right lung bronchus	Normal	28 (53.84)
	Pleural secretion	8 (15.38)
	Mucosal secretion	8 (15.38)
	Erythema	1 (1.93)
	Masslike lesion	4 (7.69)
	Cavity	0 (0)
	Stenosis	1 (1.93)
	Plaque	2 (3.85)

Left lung bronchus	Normal	29 (54.71)
	Pleural secretion	10 (18.86)
	Mucosal secretion	6 (11.32)
	Erythema	2 (3.78)
	Masslike lesion	0 (0)
	Cavity	1 (1.89)
	Stenosis	2 (3.78)
	Plaque	3 (5.66)

## Data Availability

The data are available from the corresponding author on request.

## Abbreviations

*L. blattarum*: *Lophomonas blattarum*
PCR: Polymerase chain reaction
BALF: Bronchoalveolar lavage fluid
FOB: Fiberoptic bronchoscopy
INRCL: Iranian National Registry Center for Lophomoniasis
SI: Severity index
HPFs: High-power microscopic fields
COVID-19: Coronavirus disease 2019.

## References

[1] Xue J., Li Y.-L., Yu X.-M. (2014). Bronchopulmonary infection of Lophomonas blattarum: a case and literature review. *Korean Journal of Parasitology*.

[2] Zhang X., Xu L., Wang L. L., Liu S., Li J., Wang X. (2011). Bronchopulmonary infection with Lophomonas blattarum: a case report and literature review. *Journal of International Medical Research*.

[3] Martinez-Girón R., Cornelis van Woerden H. (2013). Lophomonas blattarum and bronchopulmonary disease. *Journal of Medical Microbiology*.

[4] Fakhar M., Sharifpour A., Nakhaei M., Banimostafavi E. S., Ghasemi M., Abedian S. (2021). *Lophomonas And Lophomoniasis: Biology, Etiology, Epidemiology, Pathogenesis, Diagnosis and Treatment*.

[5] Kessel R. G., Beams H. W. (1990). Freeze fracture and scanning electron microscope studies on the nuclear envelope and perinuclear cytomembranes (parabasal apparatus) in the protozoan, Lophomonas blattarum. *Journal of Submicroscopic Cytology and Pathology*.

[6] Kilimcioglu A. A., Havlucu Y., Girginkardesler N., Çelik P., Yereli K., Özbilgin A. (2014). Putative bronchopulmonary flagellated Protozoa in immunosuppressed patients. *BioMed Research International*.

[7] Chaudhury A., Parija S. C. (2020). Lophomonas blattarum: a new flagellate causing respiratory tract infections. *Tropenmedizin und Parasitologie*.

[8] Liebler J. M., Markin C. J. (2000). Fiberoptic bronchoscopy for diagnosis and treatment. *Critical Care Clinics*.

[9] Fakhar M., Nakhaei M., Sharifpour A. (2021). Morphological and molecular identification of emerged Lophomonas blattarum infection in mazandaran province, northern Iran: first registry-based study. *Acta Parasitologica*.

[10] Martínez-Girón R., Van Woerden H. C. (2010). The burden of Lophomonas blattarum under the light microscope. *Information Display*.

[11] van Lunzen J., Altfeld M. (2014). Sex differences in infectious diseases–common but neglected. *Journal of Infectious Diseases*.

[12] Mata L. (1982). Sociocultural factors in the control and prevention of parasitic diseases. *Clinical Infectious Diseases*.

[13] Sebaa S., Behnke J. M., Baroudi D., Hakem A., Abu-Madi M. A. (2021). Prevalence and risk factors of intestinal protozoan infection among symptomatic and asymptomatic populations in rural and urban areas of southern Algeria. *BMC Infectious Diseases*.

[14] Bernin H., Lotter H. (2014). Sex bias in the outcome of human tropical infectious diseases: influence of steroid hormones. *Journal of Infectious Diseases*.

[15] Fischer J., Jung N., Robinson N., Lehmann C. (2015). Sex differences in immune responses to infectious diseases. *Infection*.

[16] Roberts C. W., Walker W., Alexander J. (2001). Sex-associated hormones and immunity to protozoan parasites. *Clinical Microbiology Reviews*.

[17] Ghafarian N., Bakhtiari E., Berenji F. (2018). The study of Lophomonas blattarum infection in children with respiratory symptoms: a descriptive clinical study in North east of Iran. *International Journal of Pediatrics*.

[18] Singhal T. (2021). Are pediatric infections with Lophomonas blattarum being missed?. *Indian Journal of Pediatrics*.

[19] Ding Q., Shen K. (2021). Pulmonary infection with Lophomonas blattarum. *Indian Journal of Pediatrics*.

[20] Saldaña N. G., Javier F. O. M., Larrauri F. R. (2017). Bronchopulmonary infection by Lophomonas blattarum in a pediatric patient after hematopoietic progenitor cell transplantation: first report in Mexico. *Journal of Thoracic Disease*.

[21] Simon A. K., Hollander G. A., McMichael A. (2015). Evolution of the immune system in humans from infancy to old age. *Proceedings of the Royal Society B: Biological Sciences*.

[22] Jiang C., Chen Q., Xie M. (2020). Smoking increases the risk of infectious diseases: a narrative review. *Tobacco Induced Diseases*.

[23] World Health Organisation (2021). Tobacco. https://www.who.int/news-room/fact-sheets/detail/tobacco.

[24] Wang H., Liu X., Umino T. (2001). Cigarette smoke inhibits human bronchial epithelial cell repair processes. *American Journal of Respiratory Cell and Molecular Biology*.

[25] Ryan D. M., Vincent T. L., Salit J. (2014). Smoking dysregulates the human airway basal cell transcriptome at COPD risk locus 19q13. 2. *PLoS One*.

[26] Sridhar S., Schembri F., Zeskind J. (2008). Smoking-induced gene expression changes in the bronchial airway are reflected in nasal and buccal epithelium. *BMC Genomics*.

[27] Berbudi A., Rahmadika N., Tjahjadi A. I., Ruslami R. (2020). Type 2 diabetes and its impact on the immune system. *Current Diabetes Reviews*.

[28] World Health Organisation (2021). Diabetes. https://www.who.int/news-room/fact-sheets/detail/diabetes.

[29] da Mota F., Murray C., Ezzat S. (2011). Overt immune dysfunction after cushing’s syndrome remission: a consecutive case series and review of the literature. *Journal of Clinical Endocrinology & Metabolism*.

[30] Hasenmajer V., Sbardella E., Sciarra F., Minnetti M., Isidori A. M., Venneri M. A. (2020). The immune system in cushing’s syndrome. *Trends in Endocrinology & Metabolism*.

[31] Verma S., Verma G., Singh D. V. (2015). Dual infection with pulmonary tuberculosis and Lophomonas blattarum in India. *International Journal of Tuberculosis & Lung Disease*.

[32] Nakhaei M., Fakhar M., Sharifpour A. (2021). First Co-morbidity of Lophomonas blattarum and COVID-19 infections: confirmed using molecular approach. *Acta Parasitologica*.

[33] Li Y., Tang X. X. (2021). Abnormal airway mucus secretion induced by virus infection. *Frontiers in Immunology*.

[34] Taheri A., Fakhar M., Sharifpour A., Banimostafavi E. S. (2022). Cavitary pulmonary lesions following emerging lophomoniasis: a novel perspective. *Respirology Case Reports*.

